# The *BAG3* gene variants in Polish patients with dilated cardiomyopathy: four novel mutations and a genotype-phenotype correlation

**DOI:** 10.1186/1479-5876-12-192

**Published:** 2014-07-09

**Authors:** Maria Franaszczyk, Zofia T Bilinska, Małgorzata Sobieszczańska-Małek, Ewa Michalak, Justyna Sleszycka, Agnieszka Sioma, Łukasz A Małek, Dorota Kaczmarska, Ewa Walczak, Paweł Włodarski, Łukasz Hutnik, Blanka Milanowska, Zofia Dzielinska, Grzegorz Religa, Jacek Grzybowski, Tomasz Zieliński, Rafal Ploski

**Affiliations:** 1 Laboratory of Molecular Biology, Institute of Cardiology, Warsaw, Alpejska 42 04-628, Poland; 2 Unit for Screening Studies in Inherited Cardiovascular Diseases, Institute of Cardiology, Warsaw, Alpejska 42 04-628, Poland; 3 Department of Heart Failure and Transplantology, Institute of Cardiology, Warsaw, Alpejska 42 04-628, Poland; 4 Department of Cardiomyopathies, Institute of Cardiology, Warsaw, Alpejska 42 04-628, Poland; 5 Department of Interventional Cardiology and Angiology, Institute of Cardiology, Warsaw, Alpejska 42 04-628, Poland; 6 Department of Pathology, Institute of Rheumatology, Warsaw, Spartańska 1 02-637, Poland; 7 The Department of Histology and Embryology, Centre of Biostructure, Medical University of Warsaw, Warsaw, Chałubińskiego 5 02-004, Poland; 8 Department of Structural Heart Diseases, Institute of Cardiology, Warsaw, Alpejska 42 04-628, Poland; 9 Department of Cardiac Surgery, Institute of Cardiology, Warsaw, Alpejska 42 04-628, Poland; 10 Department of Medical Genetics, Centre of Biostructure, Medical University of Warsaw, Warsaw, Pawinskiego 3C 02-106, Poland

**Keywords:** BAG3, Mutation, Penetrance, Dilated cardiomyopathy, Inherited heart disease

## Abstract

**Background:**

*BAG3* gene mutations have been recently implicated as a novel cause of dilated cardiomyopathy (DCM). Our aim was to evaluate the prevalence of *BAG3* mutations in Polish patients with DCM and to search for genotype-phenotype correlations.

**Methods:**

We studied 90 unrelated probands by direct sequencing of *BAG3* exons and splice sites. Large deletions/insertions were screened for by quantitative real time polymerase chain reaction (qPCR).

**Results:**

We found 5 different mutations in 6 probands and a total of 21 mutations among their relatives: the known p.Glu455Lys mutation (2 families), 4 novel mutations: p.Gln353ArgfsX10 (c.1055delC), p.Gly379AlafsX45 (c.1135delG), p.Tyr451X (c.1353C>A) and a large deletion of 17,990 bp removing *BAG3* exons 3–4. Analysis of mutation positive relatives of the probands from this study pooled with those previously reported showed higher DCM prevalence among those with missense vs. truncating mutations (OR = 8.33, P = 0.0058) as well as a difference in age at disease onset between the former and the latter in Kaplan-Meier survival analysis (P = 0.006). Clinical data from our study suggested that in *BAG3* mutation carriers acute onset DCM with hemodynamic compromise may be triggered by infection.

**Conclusions:**

*BAG3* point mutations and large deletions are relatively frequent cause of DCM. Delayed DCM onset associated with truncating vs. non-truncating mutations may be important for genetic counseling.

## Background

Dilated cardiomyopathy (DCM) is a major cause of chronic heart failure and the most common indication for cardiac transplantation [1]. In a substantial number of cases DCM is familial with autosomal dominant inheritance. Whereas a large number of genes have been shown to harbor mutations causing DCM [2] novel disease loci are continuously being reported. One interesting gene which recently has been implicated as a novel DCM locus is *BAG3*[3,4].

BAG3 belongs to a family of co-chaperones playing an anti-apoptotic role with their BAG domains binding the ATPase domain of heat shock proteins 70 (Hsc70/Hsp70) [5]. BAG3 is expressed primarily in skeletal muscle cells and cardiomyocytes, where it localizes within the Z-disc and probably acts as a signaling molecule [6]. Mice with homozygous knockout of the *BAG3* gene have degeneration of muscle fibers in striated muscle with apoptosis leading to fulminant skeletal myopathy and cardiomyopathy causing death approximately four weeks after birth [6]. *BAG3*-null zebrafish demonstrated myocardial changes resembling human DCM [3]. Since BAG3 has an anti-apoptotic activity, the DCM-associated *BAG3* mutations may act through increasing cardiomyocytes’ sensitivity to apoptosis as shown experimentally for metabolic [7] or mechanical stress [8].

A specific *BAG3* mutation virtually always occurring *de novo* (Pro209Leu) causes a severe childhood myofibrillar myopathy which is regarded as a distinct disease from that caused by other known *BAG3* mutations [9-11].

The purpose of our study was to evaluate the prevalence of *BAG3* mutations in Polish patients with DCM and to search for genotype-phenotype correlations.

## Methods

### Patients and families

**Figure 1:**
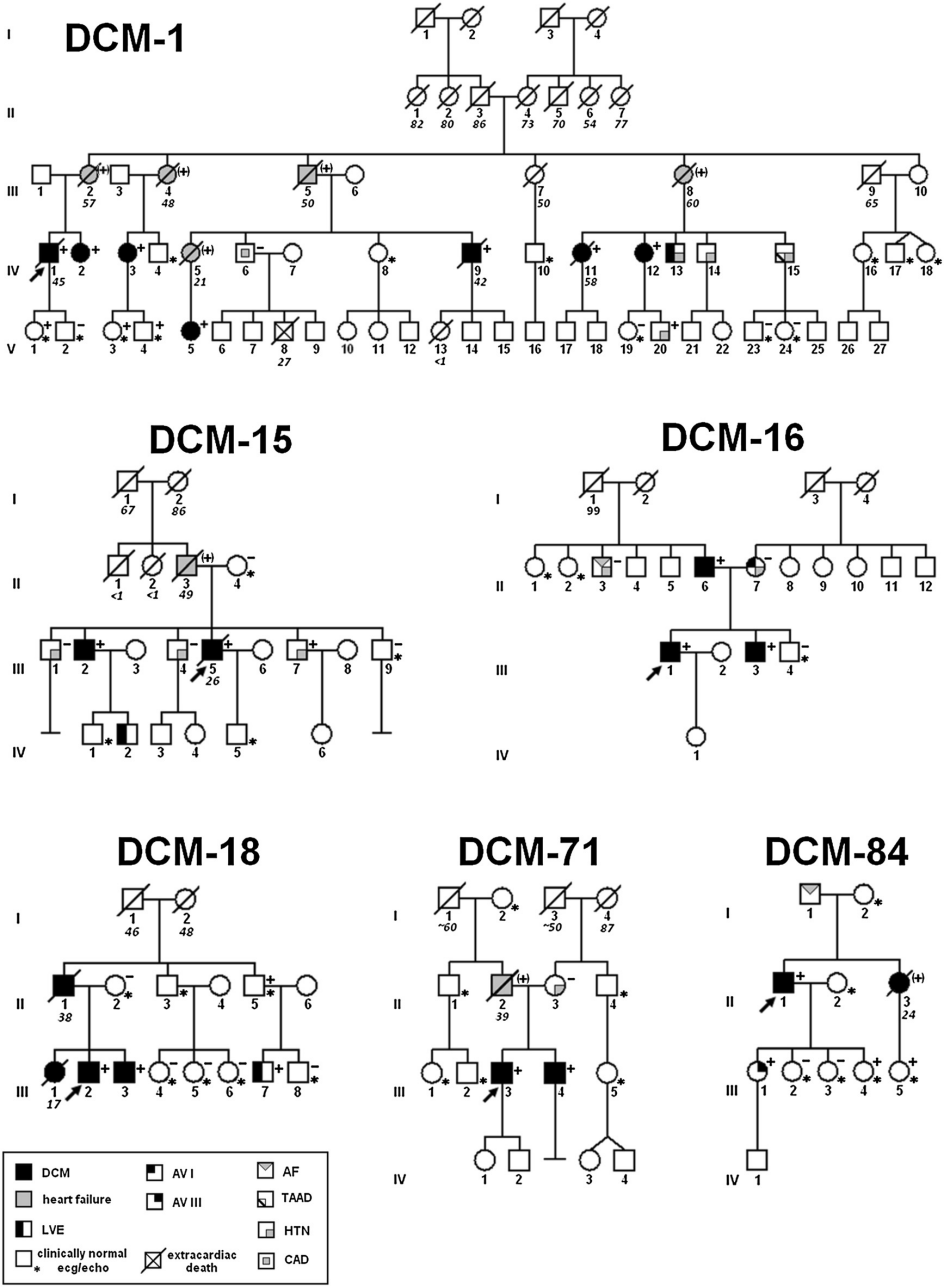
**Pedigrees of families with *BAG3 *mutations: family DCM-1 - large deletion of exons 3–4, family DCM-15 - Gly379AlafsX45, family DCM-16 - Glu455Lys, family DCM-84 - Tyr451X, family DCM-71 - Glu455Lys, family DCM-18 - Gln353ArgfsX10.** Squares represent males and circles represent females. An arrowhead denotes the proband. A diagonal line marks deceased individuals. Solid symbols denote dilated cardiomyopathy. Open symbols with asterisk denote unaffected individuals with clinically normal echo/ecg. Other features are shown in box below. The presence or absence of a *BAG3* mutation is indicated by a + or − symbol, respectively; obligate carriers are noted in parenthesis (+).

The study cohort was drawn from all index patients referred for clinical DCM genetic testing from 2010 to 2013 to the Unit for Screening Studies in Inherited Cardiovascular Diseases and involved 90 unrelated probands with DCM (67 or 74.4% male). The pedigrees of families are shown in Figure 1. DCM was diagnosed according to the ESC criteria [12] with left ventricular ejection fraction below 45% and left ventricular end-diastolic diameter exceeding 117% of value appropriate to age and body surface area. In all probands coronary angiography, or more recently coronary computed tomography angiography (CTA) was performed. Data concerning the heart transplant recipients were reviewed to confirm the diagnosis of DCM prior to heart transplantation. DCM was considered familial when more than one member was affected after clinical, electrocardiographic and echocardiographic evaluation of all the informed and consenting relatives. Creatine phosphokinase (CPK) level was obtained whenever possible. In addition, medical records of hospitalized patients were reviewed, in particular we reviewed: (1) histopathologic data of endomyocardial biopsy in two DCM patients - in one patient biopsy was performed based on clinical indications (acute onset heart failure) and in the other two pieces of endomyocardial tissue were obtained during ventricular assist device implantation due to fulminant heart failure, (2) cardiovascular magnetic resonance (CMR) data of one patient (CMR performed due to clinical suspicion of myocarditis). All patients and relatives gave written informed consent to participate in the study in accordance with the Declaration of Helsinki and study protocol was approved by the local Bioethics Committee. Once a mutation was identified adult first-degree relatives of the mutation carriers were offered mutation screening. The clinical description of the DCM-1 family was reported previously [13,14]. A phenotypic characteristic was updated to include additional family members. In one subject (III-5) from the DCM-15 family an additional permission from the Bioethics Committee was obtained to confirm the presence of mutation in the DNA extracted from myocardial tissue taken at the time of ventricular assist device (VAD) implantation.

### Screening for BAG3 mutations

DNA was extracted from the peripheral blood by phenol extraction. We screened the entire coding region together with splice sites of *BAG3* by direct Sanger sequencing. For all *BAG3* exons PCR was carried out with primers listed in Additional file 1: Table S1. The PCR conditions were: 5 min of initial denaturation at 95°C, followed by 32 cycles of 30 sec at 95°C, 55 sec at 60-68°C, 1 min at 72°C and final extension of 10 min at 72°C. PCR products were examined on 2.5% agarose gels and then sequenced using a 3500×L Genetic Analyzer (Applied Biosystems, Foster City, CA, USA) and BigDye Terminator v3.1 Cycle Sequencing Kit (Applied Biosystems) according to the manufacturer’s instructions. The results were analyzed with Variant Reporter 1.1 Software (Applied Biosystems).

### Screening for large deletions in BAG3

The screening for large deletions in *BAG3* was performed by quantitative PCR (qPCR) using Applied Biosystems 7500 Real Time PCR System and MESA GREEN MasterMix Plus, Low ROX (Eurogentec, Belgium). The PCR conditions were: 10 min of initial denaturation at 95°C, followed by 40 cycles of 15 sec at 95°C and 1:45 min at 60°C. Albumin gene (*ALB*) was used as a reference. All qPCR analyses were run in duplicates. Copy number was calculated by delta delta C_t_ method i.e. ∆∆C_t_ = (C_t BAG3_ - C_t ALB_)_test_ - (C_t BAG3_ - C_t ALB_)_wild type_ where C_t_ denotes mean cycle number in which threshold value of fluorescence was recorded for the *BAG3* or *ALB* primers for the reference (a sample without deletion, i.e. ‘wild type’) and tested (‘test’) sample, respectively. For C_t_ determination the default method available on the instrument was used. ∆∆C_t_ > 0.8 was regarded as indicative of deletion. Subsequent fine mapping of the detected deletion was performed in a similar way using a stepwise approach. The sequences of *BAG3* and *ALB* primers used at both stages of analysis are listed in Additional file 2: Table S2. Primers used for final PCR-amplification and sequencing of the breakpoint region were: BAG3intron2/3 5′TGC TCT CAA TTT CGA GGT GA 3′ and BAG3del 5′ CGG GAG AAT CAT GAG GTC AG 3′ (the forward and the reverse primer, respectively). The same primers as those used for sequencing of the breakpoint region were also used for screening of family members for the presence of the deletion.

### Statistical analysis

Statistical significance of difference in DCM prevalence among subjects with truncating vs. non-truncating *BAG3* mutations was performed by Chi square test. Age at DCM onset in these groups was analyzed by Kaplan-Meier survival curves and Cox’s F-Test. All analyses were performed using Statistica software package (StatSoft).

## Results

### Analysis of probands

**Figure 2:**
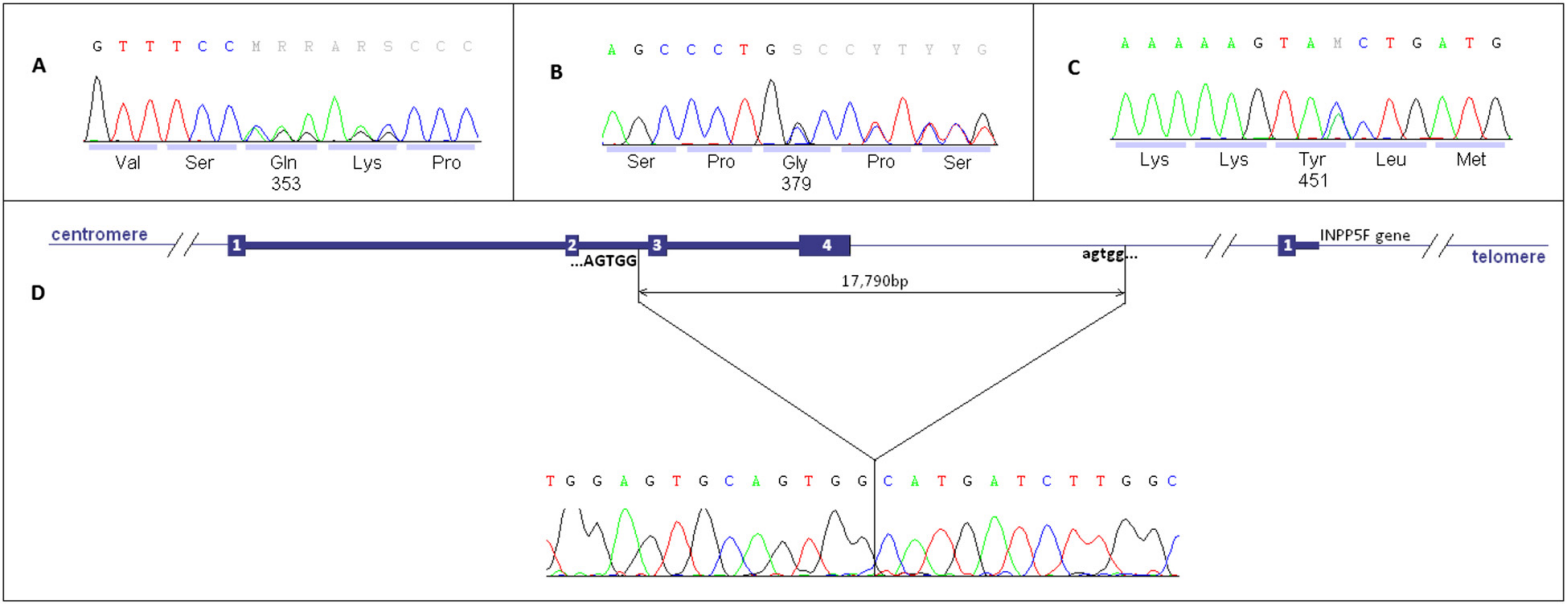
**Chromatograms illustrating novel *BAG3 *mutations found. A** – p.Gln353ArgfsX10 (c.1055delC), **B** - p.Gly379AlafsX45, (c.1135delG), **C** - p.Tyr451X (c.1353C>A), **D** - large deletion of 17,990 bp removing *BAG3* exons 3–4, chromatogram shows breakpoint sequence.

Direct sequencing of the *BAG3* coding sequence and splice sites showed four different mutations in five DCM families: known missense Glu455Lys mutation in 2 families (DCM-16 and DCM-71), two novel small deletions predicted to cause frameshifts: Gln353ArgfsX10 (c.1055delC) (DCM-18), Gly379AlafsX45 (c.1135delG) (DCM-15), and a novel Tyr451X (c.1353C>A) mutation predicted to insert a premature stop codon (DCM-84). Each of the truncating mutations occurred in a single family. Chromatograms illustrating the novel mutations are shown in Figure 2. None of the detected *BAG3* mutations were found in population databases.

To search for large deletions in *BAG3* we performed qPCR comparing dosage of DNA within *BAG3* exons with that a reference gene (albumin). In one sample (proband from the DCM-1 family) using primers RT_Exon3 and RT_Exon4.1 located in exons 3 and 4 respectively we observed shifts in amplification curves consistent with presence of a heterozygous deletion (∆∆C_t_ = 0.92).

Next, we carried out qPCR experiments aimed at stepwise narrowing of the deleted region. Eight such experiments were performed and they eventually allowed to narrow down the deletion to a region sufficiently short to design PCR primers likely to amplify the deletion breakpoint. The PCR product was obtained and its identity was verified by sequencing which also demonstrated that the deletion encompassed a region of 17,990 bp including 3 and 4 exons of *BAG3* and extending into the telomeric end in the direction of the *INPP5F* gene (Figure 2D). We noted that the 5 bp of the DNA sequence directly adjacent to the centromeric end of the deleted fragment was identical to the sequence at the telomeric end of the deleted fragment (AGTGG in both cases, Figure 2D).

The prevalence among probands of all detected *BAG3* mutations was 6/90 or 6.7% with 95% confidence interval from 3.1% to 13.8%.

### Clinical and histopathological findings among the probands

Among the six probands with *BAG3* mutations one had an acute onset with a fulminant course of the disease (DCM-15 III-7) and died awaiting heart transplantation (HTX) while on ventricular assist devices within three months from the diagnosis. Three probands (DCM-18 III-2, DCM-1 IV-1, DCM-71 III-4) received HTX after 60, 108 or 196 months from the diagnosis, respectively. Two probands experienced partial recovery: one remains stable after 108 months with LVEF 36% and another one with 2 months follow-up’ time experienced significant improvement in LV systolic function (LVEF 51%) while on standard treatment of heart failure. Clinical characteristics of probands are given in Additional file 3: Table S3.

Histopathological analysis in patients with the Glu455Lys variant (DCM-16 III-1) was performed within one month after the onset of acute heart failure. The findings were non specific with features of cellular hypertrophy, myocardial cell degeneration and interstitial fibrosis as usually found in DCM. In the specimen from the patient with the Gly379AlafsX45 variant (DCM-15 III-5) tissue severe myocytolysis with scarce inflammatory infiltrate was found.

### Analysis of family members – clinical findings

In addition to six probands we have identified 21 relatives with *BAG3* mutations. Eleven (52.4%) of these subjects had DCM with the mean age at onset of 36 years (range 15–53). There were two deaths: one female died suddenly at 58 years after 60 months from the diagnosis and there was one heart failure death at 42 years after 120 months from the diagnosis in a male patient. Significant left ventricular dysfunction at the end of follow-up with LVEF ≤ 45% was present in 9 relatives (42.9%). In two mutation carriers (9.5%) an improvement in LV function was observed over the follow-up while on standard treatment for heart failure. Persistent normal LV function was found in 10 (47.6%) carriers whose mean age was 29.2 years (range 20–53). Clinical characteristics of relatives with *BAG3* mutations are given in Additional file 3: Table S3.

CMR study in DCM-15 III-2 (Gly379AlafsX45), performed within one month after the onset of symptoms showed dilated hypocontractile left ventricle and the presence of CMR diagnostic criteria for myocarditis [15]. In particular, T2-weighted images demonstrated global myocardial signal intensity (SI) increase (>2) in comparison to skeletal muscle indicating global edema (Additional file 4: Figure S1A). There was also an increased global myocardial early gadolinium enhancement ratio (>4) in comparison to skeletal muscle in* gadolinium-enhanced T1-weighted images indicating global myocardial hyperemia. Late gadolinium enhancement (LGE) revealed intramyocardial foci in the interventricular septum and at the low junction point of the right and left ventricle indicating myocardial fibrosis (Additional file 4: Figure S1B). At the time of CMR study, serological examinations showed positive serum IgM Lyme titres and borderline IgG, positive IgG antyParvovirus B19 titres and detectable human herpes virus 6 (HHV6) genome in the serum. The patient received gammaglobulin 0.1 g/kg i.v. along with doxycycline 100 mg PO bid for 21 days leading to substantial clinical improvement (a rise in LVEF up to 36%).

### Analysis of family members – genotype dependent penetrance of BAG3 related DCM

All subjects with *BAG3* mutations who were DCM free had truncating mutations whereas all those with missense mutations were affected (Table 1). A trend for higher DCM prevalence among those with missense *BAG3* mutations was also found on reanalysis of data previously reported for mutation positive family members of probands with *BAG3* related DCM [3,4]. When our data were combined with data reported previously [3,4] there was a statistically significant difference indicating higher prevalence of DCM among those with non-truncating vs. truncating *BAG3* mutations (OR = 8.33, P = 0.045, Table 1). The association was even stronger (P = 0.0058) when a healthy child aged 7 reported by Villard et al. was excluded from analysis as uninformative due to young age (Table 1).

**Table 1:** **Presence of DCM among subjects with truncating and non-truncating *BAG3 *** **mutations (probands excluded)**

	**Truncating *BAG3 *** **mutations**	**DCM**	**Normal**
		**N (%)**	**N (%)**
This study	Yes	8 (44)	10 (56)
	No	3 (100)	0
Previous studies	Yes	19 (79)	5 (21)
	No	12 (92)	1 (8)
Combined*	Yes	27 (64)	15 (36)
	No	15 (94)	1 (6)

*OR = 8.33, P = 0.045 (P = 0.0058 after excluding a DCM free 7 year old child as non-informative, Fisher exact test), OR calculated for presence of DCM among non-truncating vs. truncating BAG3 mutations.

**Figure 3:**
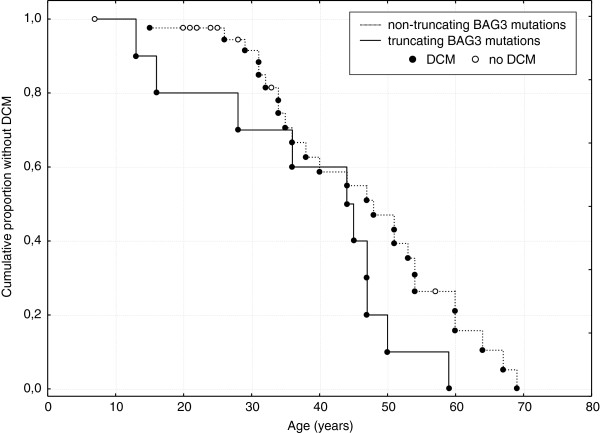
**Figure Kaplan-Meier survival curves for relatives of probands with truncating and non-truncating *BAG3 *mutations.** (P = 0.006, Cox’s F-Test, data from the present study pooled with data from 2 previous studies [3,4]).

These results suggested that missense vs. truncating *BAG3* mutations could be associated with earlier disease onset and/or lower penetrance or just age difference between the groups. To study this further we analyzed age at DCM onset in the two groups by Kaplan-Meier survival analysis. Figure 3 shows time to DCM onset in family members of the probands stratified by the mutation category (subjects from the present study were pooled with those reported previously [3,4]). As can be seen, whereas by the age of 70 the disease penetrance is apparently 100%, by the age of 50 years the prevalence of DCM among those with non-truncating vs. truncating *BAG3* mutations differs (~90% vs. ~55%, respectively, Figure 3). The difference between the two groups in DCM free survival was statistically significant (P = 0.006, Cox’s F-Test).

## Discussion

While studying a cohort of 90 adult unrelated DCM patients and their relatives we found *BAG3* mutations in 6 probands and 21 family members. Four of the observed mutations were novel: Gln353ArgfsX10 (c.1055delC), Gly379AlafsX45, (c.1135delG), Tyr451X (c.1353C>A) and a large deletion removing 17,990 bp. Analysis of affection status in *BAG3* mutations carriers among relatives of the probands from our cohort together with those reported previously [3,4] showed difference in age related penetrance which, interestingly, suggested later onset of disease in those with non-truncating vs. truncating mutations.

The *BAG3* Glu455Lys (rs397516881) has been reported previously as pathogenic although this was based on a single report [3,4] and the ClinVar database [16] describes the variant as having uncertain significance. The conclusion on Glu455Lys pathogenicity was based on a single family in which this variant was found in five individuals four of whom had DCM [3,4]. Our finding of rs397516881 in two apparently unrelated DCM probands and three affected family members together with its lack in NHLBI GO exome sequencing project (ESP) [17], 1000 genomes databases [18] and our in-house exome database of 250 Poles argues for genuine association of *BAG3* Glu455Lys with DCM. However, this conclusion would certainly be strengthened by additional data from other populations.

The Gln353ArgfsX10 (c.1055delC), Gly379AlafsX45 (c.1135delG) and the 17,990 bp deletion removing exons 3–4 are likely to be pathogenic as they are predicted to remove a larger C terminal part of the *BAG3* protein than the two previously reported pathogenic *BAG3* mutations: R395GfsX48 and S385QfsX56 [4]. This argument does not apply to Tyr451X which is the most C terminal (the least truncating) *BAG3* mutation reported so far. However, Tyr451X is also likely to be pathogenic as it removes more than half of the single BAG domain present in the *BAG3* protein. The deleted part contains whole alpha3 helix and a significant part of the alpha2 helix, both of which are responsible for binding between *BAG3* and Hsc710/Hsp70 [19]. The part of alpha2 and the whole alpha3 helix which are deleted by Tyr451X mutation contain numerous aminoacids highly conserved both among BAG domains of different human BAG proteins [19] and between BAG3 proteins of different species (Additional file 5: Figure S2). Finally, the *BAG3* Tyr451X mutation, similar as Gln353ArgfsX10 (c.1055delC), Gly379AlafsX45 (c.1135delG), has not been observed in NHLBI GO exome sequencing project (ESP) [17], 1000genomes database [18], ClinVar databases [16] or our in-house exome database of 250 Poles. Whereas all these findings suggest pathogenicity, as recently emphasized for other variants [20,21], more data is needed for a firm conclusion.

Among the novel B*AG3* mutations the most interesting is the large deletion of 17,990 bp which removes exons 3–4 and a chromosome fragment extending in the direction of the *INPP5F* gene. This deletion, together with the deletion of 8,733 bp described by Norton et al. [3] suggests that the 3′ part of the *BAG3* locus may be prone to structural rearrangements. The 17,990 bp deletion probably originated due to microhomology at the breakpoints and thus may be recurrent [22]. These observations highlight the necessity for screening *BAG3* for copy number variations (CNV) variants in addition to point mutations.

The prevalence of *BAG3* defects in our cohort was relatively high (6/90 or 6.7%) being comparable to the prevalence of mutations in *LMNA* (~6%) which has been regarded as the most frequently mutated locus in DCM [23,24]. Thus, the *BAG3* gene emerges as a major DCM locus. Although its role is clearly smaller than that of *TTN*, whose mutations have recently been shown to occur in up to 25% of DCM patients [25] our results indicate that, at least in Polish population, a systematic screening of *BAG3* should be offered to DCM patients.

The findings that truncating *BAG3* mutations cause disease with later onset than missense variants may be important for genetic counselling. Although it should be confirmed by a study specifically addressing disease severity, our results suggest that missense *BAG3* mutations may have a stronger pathogenic effect than the truncating variants. Interestingly, that would contrast with observations for the *LMNA* gene whose truncating variants were recently associated with a more severe DCM [26]. As shown for the *LMNA* gene [27], a likely main effect of truncating mutations is the loss of function, whereas missense variants may in addition (or alternatively) exert dominant negative effects. Pathogenicity of *BAG3* haploinsufficiency is supported by DCM association shown for a number of truncating variants, in particular the severely truncating *BAG3* Arg90X mutation [3]. However, earlier onset of DCM suggestive of a more severe phenotype associated with non-truncating mutations found in our study indicates that at least some *BAG3* missense variants exert dominant-negative effects. This notion is consistent with a distinct and severe phenotype (childhood onset myopathy) consistently observed in patients with the *BAG3* p.Pro209Leu mutation [9-11].

Clinical data from our study suggest that in presence of *BAG3* defects stress may trigger acute onset DCM with hemodynamic compromise, which is consistent with *in vitro* studies implicating *BAG3* in the control of apoptosis and response to stress stimuli [7,8]. A *BAG3* mutation carrier with history of acute heart failure (DCM-15 III-2) fulfilled CMR diagnostic criteria for myocarditis with serological evidence of acute Lyme disease and past Parvovirus B19 infection, and a detectable HHV6 genome in the blood. Intramyocardial foci of late gadolinium enhancement in the interventricular septum were previously observed in patients with myocarditis associated with human HHV6 infection or Lyme disease [12,28-30]. Moreover, all severely affected *BAG3* mutation carriers of the DCM-18 family had disease onset related to the influenza of 1988 along with two of deceased first-degree members of the family who developed progressive heart failure leading to death. Furthermore, all subjects with *BAG3* mutations who had acute onset of heart failure following viral-like illness had very low LVEF (10-22%), that is consistent with poor response to any pathogen-related stress.

## Conclusions

In conclusion, by studying Polish patients we found that *BAG3* mutations are relatively frequent cause of DCM. We report four novel pathogenic *BAG3* variants including a large deletion and show, for the first time, that truncating *BAG3* variants are associated with DCM characterized by later onset than missense variants. Clinical data from our study suggest that in *BAG3* mutation carriers infection may trigger acute onset DCM with hemodynamic compromise. Despite the limitations of our study such as relatively small number of subjects and a retrospective design our results add to the knowledge on *BAG3* related diseases and, if replicated in additional cohorts, may be important for genetic counseling.

## Competing interests

The authors declare that they have no competing interests.

## Authors’ contributions

MF: performing the experiments (DNA sequencing and quantitative PCR analysis), analysis and interpretation of data, critical revision of the manuscript. ZTB: design of the study, analysis and interpretation of data, drafting manuscript, critical revision of the manuscript content; MSM: acquisition of clinical data of transplanted patients and end-stage dilated cardiomyopathy patients, analysis of data; EM: acquisition of clinical data, analysis of data; JS: acquisition of clinical data, analysis of data; AS: acquisition of clinical data, analysis of data; ŁAM: acquisition of MRI data, analysis of data, critical revision of the content; DK: acquisition of clinical data, analysis of data; EW: acquisition of histopathologic data, critical revision of the content; PW: design of the study, analysis of data, critical revision of the manuscript; ŁH: design of the study, analysis of data, critical revision of the manuscript; BM: acquisition of clinical data, analysis of data; ZD: acquisition of data, analysis of data; GR: acquisition of clinical data of patients on mechanical cardiac support, biopsy specimen handling, analysis of data; JG: taking part in the design of the study, analysis and interpretation of data, critical revision of the content; TZ: taking part in the design of the study, analysis and interpretation of data, critical revision of the content; RP: design of the study, performed statistical analysis, handling funding and supervision of DNA sequencing and quantitative PCR analysis, writing the paper. All Authors read and approved the final manuscript.

## Supplementary Material

Additional file 1: Table S1 Primers used for mutation screening of *BAG3* coding sequence and the splice sites. Click here for file

Additional file 2: Table S2 Primers used for screening for large *BAG3* deletions **(A)** and for subsequent fine mapping of the deletion region (**B**, listed in order of use). Click here for file

Additional file 3: Table S3 Clinical characteristics of probands and relatives. Description of data: Legend: Fam - Family; Sub - Subject; A,g - Age at genetic inquest; Sex - sex (M-male, F-female); R - Relatives (1-1st degree, 2-2nd degree, 3-3rd degree); A,d - Age at diagnosis/at screening; Phen – phenotype (DCM/ N-normal heart); EF – LVEF; N,os - NYHA at onset of symptoms/at screening; E/a - ECG/arrhythmia; T,d - Time from diagnosis (months); EDD,f-p - LVEDD (mm) at last follow-up; EF,f-p - LVEF (%) at last follow-up; I/P - ICD/PM; Additional – comments/co-existing disease/family history; (*) - DNA available, died earlier; (†) - DNA extracted from the endomyocardial tissue; ACE-I - angiotensin converting enzyme inhibitors; AII - angiotensin II blockers; a-v - atrio-ventricular; BB - beta-blockers; DOE – dyspnea on effort; ECG - standard 12-lead electrocardiogram; FA/VA - atrial fibrillation/atrial flutter; HF - heart failure; HTN - hypertension; HTX - heart transplantation; ICD - internal cardioverter defibrillator; LBBB - left bundle branch block; LV - left ventricular; LVEDD - left ventricular enddiastolic dimension; LVEF - left ventricular ejection fraction; MI - myocardial infarction; nsVT - nonsustained ventricular tachycardia; P - palpitations; PM - pacemaker, QTc - corrected QT interval; SCD - sudden cardiac death; SR - sinus rhythm; VAD - ventricular assist device; VEX - ventricular extrasystole). Click here for file

Additional file 4: Figure S1 Cardiovascular magnetic resonance images demonstrating signs of myocarditis in patient DCM-15 II-2. Description of data: T2-weighted image in short axis demonstrating global myocardial edema (increased signal intensity of the myocardium in comparison to the skeletal muscle), B) short axis image showing intramyocardial foci of late gadolinium enhancement (arrows). Click here for file

Additional file 5: Figure S2 The highly conserved sequence of *BAG3* exon 4 of different species with marked BAG domain. Description of data: Black arrow indicates the position of Tyr451X mutation. The color saturation reflects the conservation of amino acid. The red frame outlines human BAG3 protein ID ENSP00000358081. Click here for file
