# Supplementary figures and images for: The BAG3 gene variants in Polish patients with dilated cardiomyopathy: four novel mutations and a genotype-phenotype correlation
Source: J Transl Med. 2014 Jul 9;12:192. doi: 10.1186/1479-5876-12-192 (PMC4105391; doi:10.1186/1479-5876-12-192)

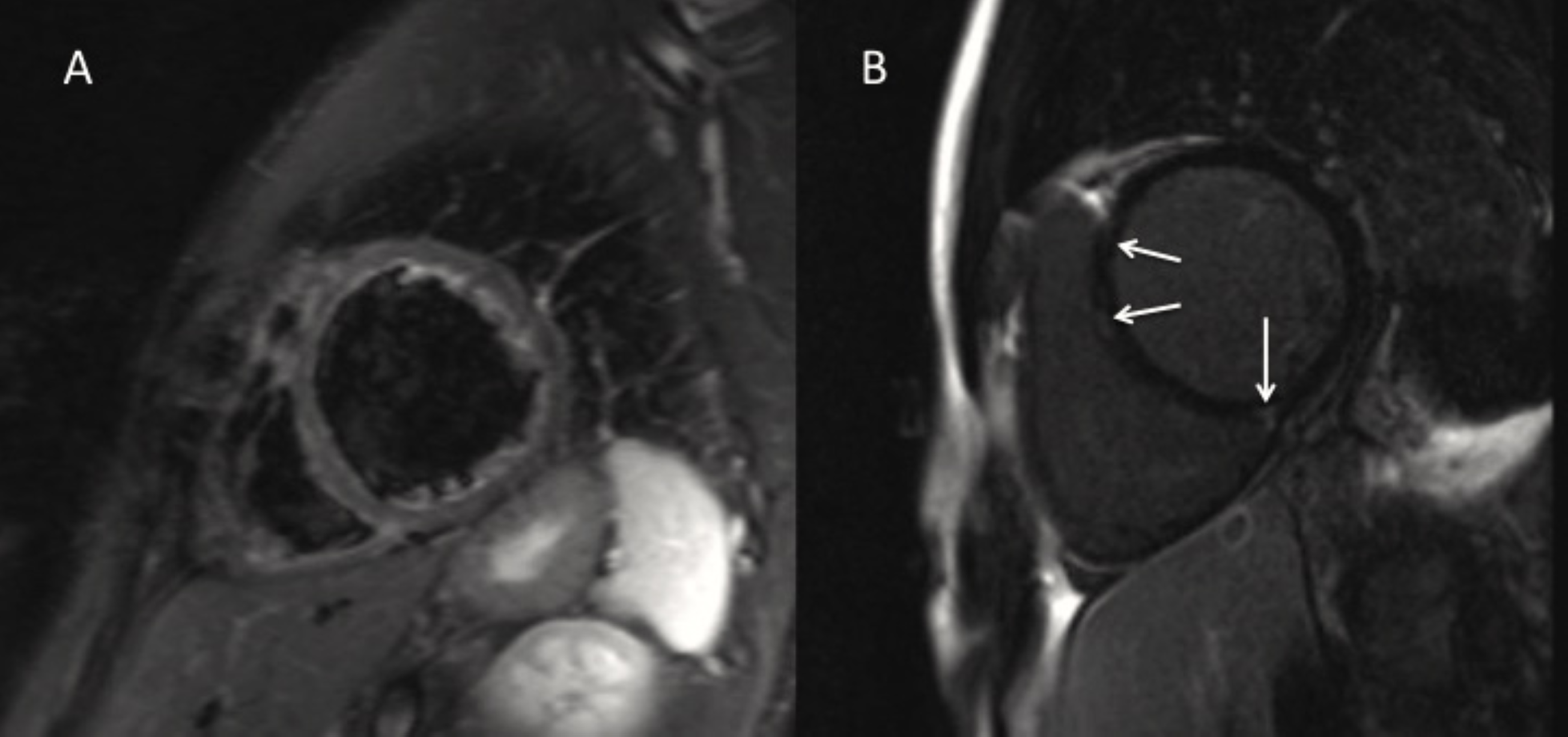

Supplement: Additional file 4: Figure S1 — Cardiovascular magnetic resonance images demonstrating signs of myocarditis in patient DCM-15 II-2. Description of data: T2-weighted image in short axis demonstrating global myocardial edema (increased signal intensity of the myocardium in comparison to the skeletal muscle), B) short axis image showing intramyocardial foci of late gadolinium enhancement (arrows). [file 1479-5876-12-192-S4.tiff]

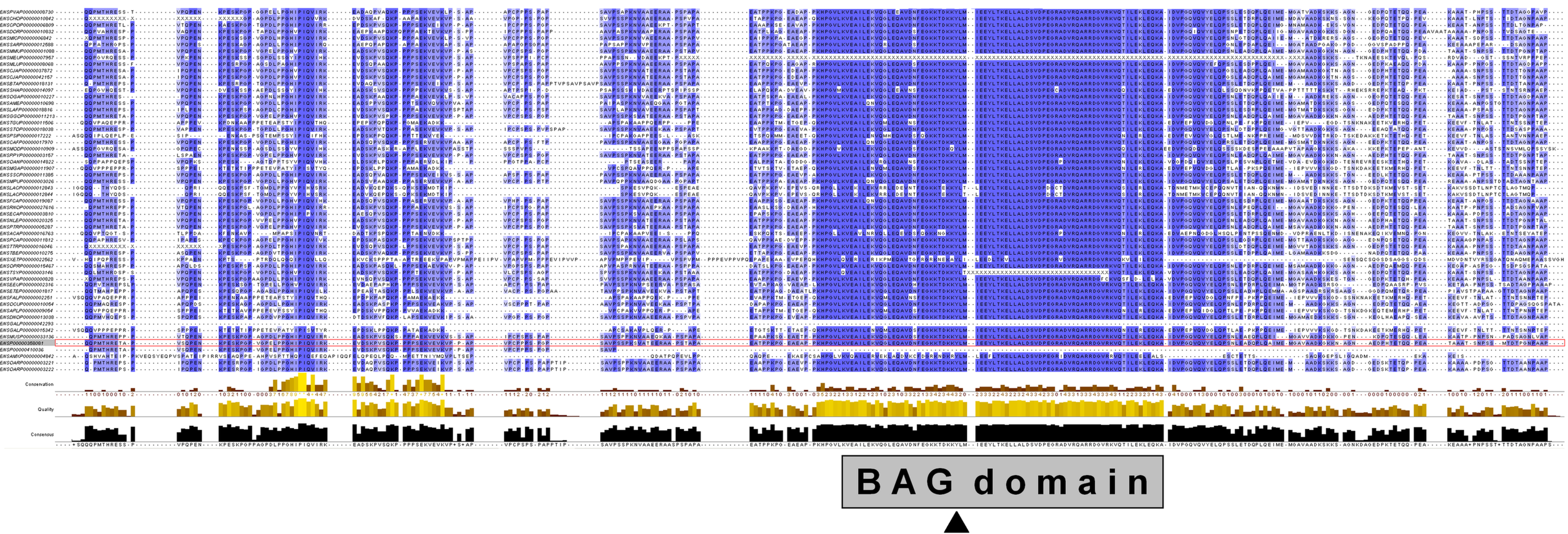

Supplement: Additional file 5: Figure S2 — The highly conserved sequence of BAG3 exon 4 of different species with marked BAG domain. Description of data: Black arrow indicates the position of Tyr451X mutation. The color saturation reflects the conservation of amino acid. The red frame outlines human BAG3 protein ID ENSP00000358081. [file 1479-5876-12-192-S5.tiff]
